# Inhibition of Quorum Sensing and Virulence Factors of *Pseudomonas aeruginosa* by Biologically Synthesized Gold and Selenium Nanoparticles

**DOI:** 10.3390/antibiotics10121461

**Published:** 2021-11-27

**Authors:** Soha Lotfy Elshaer, Mona I. Shaaban

**Affiliations:** Department of Microbiology and Immunology, Faculty of Pharmacy, Mansoura University, Mansoura 35516, Egypt; dr_sohaloftyeldamarawy@mans.edu.eg

**Keywords:** Se nanoparticles, Au nanoparticles, quorum sensing inhibition, virulence factors, antimicrobial, *P. aeruginosa*

## Abstract

The development of microbial resistance requires a novel approach to control microbial infection. This study implies the microbial synthesis of nanometals and assessment of their antivirulent activity against *Pseudomonas aeruginosa*. *Streptomyces* isolate S91 was isolated from soil with substantial ability for growth at high salts concentrations. The cell-free supernatant of S91was utilized for the synthesis of Au-NPs and Se-NPs. The 16S rRNA sequence analysis of *Streptomyces* S91 revealed that S91 had a high similarity (98.82%) to *Streptomyces olivaceous.* The biosynthesized Au-NPs and Se-NPs were characterized using a UV-Vis spectrophotometer, dynamic light scattering, transmission electron microscopy, energy dispersive X-ray diffraction and Fourier-transform infrared spectroscopy. The quorum sensing inhibitory (QSI) potential of Au-NPs and Se-NPs and the antivirulence activity was examined against *P. aeruginosa*. The QSI potential was confirmed using RT-PCR. The synthesized Au-NPs and Se-NPs were monodispersed spherical shapes with particle size of 12.2 and 67.98 nm, respectively. Au-NPs and Se-NPs eliminated QS in *P. aeruginosa* at a concentration range of 2.3–18.5 µg/mL for Au-NPs and 2.3–592 µg/mL for Se-NPs. In addition, Au-NPs and Se-NPs significantly inhibited QS-related virulence factors, such as pyocyanin, protease and, elastase in *P. aeruginosa*. At the molecular level, Au-NPs and Se-NPs significantly suppressed the relative expression of QS genes and toxins. Hence, the biosynthesized Au-NPS and Se-NPS could be substantial inhibitors of QS and virulence traits of *P. aeruginosa*.

## 1. Introduction

The spread of antibiotic-resistant bacteria, both in hospitals and in community-acquired infections, is one of the major problems of antibiotic therapy [1]. The emergence and continuous outbuilding of new resistance mechanisms dismiss the effort of medical staff to treat infectious diseases, resulting in high rates of mortality [2]. Cephalosporine, quinolone, and aminoglycoside resistance are widespread, and most antimicrobials have become ineffective in treating resistant isolates [3]. Resistance to colistin, a last resort treatment, has been reported globally, increasing the risk of infections caused by untreatable bacteria. Antibiotics that are currently available are ineffective against pandrug-resistant pathogens, and antibiotic combinations are required in systemic infections. In addition, the development of resistance to antimicrobial agents forces the administration of high doses of antibiotics, with adverse and toxic effects. This strengthens the need for novel therapeutic drugs [4].

Moreover, most pharmaceutical companies have stalled their investment in the development of new antibiotics. Once antibiotics are produced, bacterial resistance rapidly develops within a short period of time, and profits are prematurely curtailed [1].

Additionally, most bacteria can develop virulent strategies, such as biofilm formation, production of many degrading enzymes, and microbial toxins that assess microbial dissemination and infection. *Pseudomonas* biofilm is considered one of the main virulence factors associated with *Pseudomonas* infection as the organism can form exopolysaccharide that cannot not be penetrated with antimicrobial agents [5]. Additionally, bacteria develop cell–cell communication behavior called quorum sensing (QS) which controls an arsenal of virulence factors [6]. This is achieved through the secretion of signaling molecules called autoinducers. Acyl homoserine molecules are considered the backbone structure of the signal molecules produced by Gram-negative bacteria, such as *Pseudomonas aeruginosa.* QS circuit in *P. aeruginosa* is maintained through a series of signals and regulatory genes including 3-oxododecanoyl L-homoserine lactone, butanoyl homoserine lactone, 2-heptyl-3-hydroxi-4-quinolone and 2-(2-hydroxyphenyl) thiazole-4-carbaldehyd which are under the control of lasI/R, rhlI/R, pqsABCDE/pqsR and AmbBCDE/IqsR regulatory systems, respectively [7,8,9,10]. Targeting QS in humans becomes attractive as no homologs to known QS components exist in humans. In addition, the prevention of bacterial QS could control the pathogenesis and dissemination of *P. aeruginosa.* Consequently, shifting the focus from antibacterial to nano-antibiotics/nano anti-QS may reveal a new strategy for combating microbial virulence and resistance [11,12,13].

The progress in nanotechnology with promising applications in medicine provides a novel approach for combating microbial resistance. Nano-metals with novel modes of action could provide a solution to eliminate microbial infections [14]. The nanometals are characterized by ultra-small particle size and large surface area, making them of great interest in the treatment of bacterial infections [15]. The antimicrobial activity of nanometals is multifactorial; they disturb cellular permeability, causing cytoplasmic leakage and microbial damage [16]. They enhanced the potential for SH binding, hence, disrupting protein synthesis and microbial propagation [15]. In addition, nano-preparations have a high affinity for DNA binding with elimination of microbial replication [17]. Furthermore, the release of reactive oxygen species oxidizes the cell components, causing cellular damage.

Metal and metal oxide nanoparticles are now commonly used in a variety of medical and pharmaceutical applications. Au-NPs and Se-NPs are two medically significant nanometals. Au-NPs have high physical and chemical stability, as well as high biocompatibility. Au-NPs are used in a variety of medical applications such as gene, and drug delivery, photothermal, photodynamic, and radiation therapy, as well as various biological applications [18]. Se-NPs are non-toxic, and exhibit low cytotoxicity compared to their salts, selenite (SeO_3_^2−^) and selenate (SeO_4_^2−^). Se-NPs exhibit potential therapeutics in various inflammatory disorders, such as nephropathy, arthritis, diabetes, and cancer. Se-NPs are considered as safe carriers of various drugs to the site of action. The bioactivity of Se-NPs includes antioxidant activity, pro-oxidant effects, and anticancer effects [19,20,21]. New antivirulence approaches have been developed as a potential target for fighting microbial infections. Several studies were conducted to evaluate the antivirulence properties of various quorum sensing inhibitors (QSIs) and antibiofilm agents [22]. However, the antivirulence activities of nanometals have not been widely investigated. Targeting bacterial virulence using nanometals could assist in the management of microbial pathogenesis, and dissemination of microbial pathogens.

There are various approaches for the synthesis of nanometals: (i) a physical approach; (ii) chemical approach; and (iii) biological approach. Physical and chemical synthesis methods are cheap and provide high yield, nevertheless, these methods include the use of lethal solvents, and precursor [23]. In the biological methods, the metal ions are reduced to nanometals using biological agents. Plant extracts, intracellular or extracellular extracts of fungi and bacteria have been used for reduction of metals to nanoform [24].

With the promise of nanometals, we are interested in the microbial synthesis of nanometals as an efficient and safe method. Previous studies have focused on the antibacterial potential of soil microbiota [25,26]. Investigations were restricted to whether the microorganism isolated from soil could kill or inhibit the growth of bacteria either Gram-positive or Gram-negative pathogens [27] through the production of antimicrobial agents. In addition, *Streptomyces* can produce immune suppressive, antitumor agents, various enzymes and secondary metabolites [28]. However, the exploration of *Streptomyces* for biosynthesis of nanometals has not been widely studied and could be used for the biological synthesis of nanometals.

This study aims to isolate *Streptomyces* from soil and screen them for the ability to biosynthesize nanometals. The produced nanometals were evaluated by UV-Vis spectroscopy, particle size analysis, Zeta potential, transmission electron microscope (TEM) photography, energy dispersive X-ray diffraction (EDX) and Fourier-transform infrared spectroscopy (FTIR). This could provide a potential target for the development of novel therapeutic agents and permit a strategy for the synthesis of nanometals with antimicrobial and antipathogenic effects.

## 2. Results

### 2.1. Isolation and Purification of STREPTOMYCES

A total of twenty-five actinomycetes isolates were purified from eight soil samples; six agriculture soils were collected from the Dakahlia governorate, and two sand soils were collected from the Damietta governorate (Supplementary Table S1). The isolates exhibited rapid, moderate to abundant growth on ISP2 media. All samples showed profuse sporulation on solid media. The isolates were characterized by their leathery, tough, pigmented colonies and filamentous growth. The purified colonies were stained with Gram stain and examined under the microscope. *Streptomyces* had a branched network of mycelia with conidiophores at the ends of the aerial mycelia.

### 2.2. Synthesis of Nanometals

*Streptomyces* isolates S1, SS2, S91, S29B, and S41.1 can grow in the presence of a high salt concentration of 60 mM selenium oxide (SeO_2_) and 3 mM of chloroauric acid (HAuCl_4_). *Streptomyces* S1, SS2, S91, S29B, and S41.1 were selected and propagated on the ISP2 agar medium, and the plates were incubated at 30 °C for 10–14 days. *Streptomyces* pure colonies were picked and maintained on new ISP2 plates at 30 °C. Spore suspension was prepared in 50% glycerol (*v*/*v*) and stored at −80 °C.

Au-NPs were synthesized by isolates S1, SS2, S91, S29B, and S41.1 with λ_max_ 0.17, 0.59, 1.17, 2, and 1.7 at 641, 583, 520, 536, and 582 nm, respectively. Se-NPs were not formed by the tested supernatants within 24 h. After 48 h of treatment, the supernatant of isolate S91 reduced selenium oxide into Se-NPs with a maximum of 1.1 at 450 nm. Therefore, isolate S91 was selected for the synthesis of nanoscale Au-NPs and Se-NPs for 48 h (Supplementary Table S2).

### 2.3. Characterization of Nanometals

#### 2.3.1. Color Change and UV-Vis Spectra Analysis

**Figure 1:**
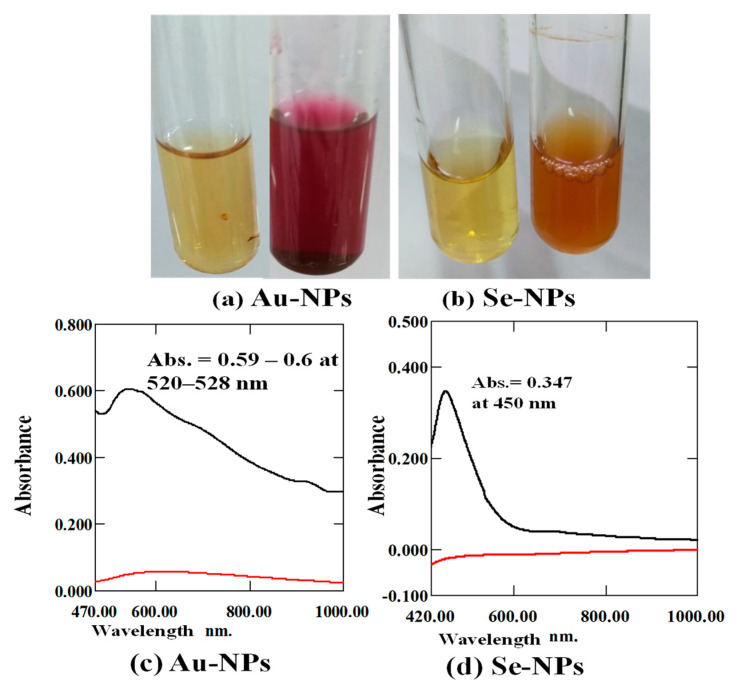
Color change and UV-Vis spectra analysis: distinct colors of the biological synthesized nanometals (**a**) Au-NPs with purple color and (**b**) Se-NPs with orange color. UV-Vis spectrum analysis of the diluted biosynthesized nanometals (**c**) Au-NPs and (**d**) Se-NPs (black line) and the cell free supernatant as negative control (red line).

The reduction of metal ions into nanometals was monitored by examination of the color change during the incubation period. It was noticed that the color of the cell-free supernatant was converted into purple and orange, which represents a primary indication of the reduction of the ions and the formation of Au-NPs (Figure 1a), and Se-NPs (Figure 1b), respectively.

UV-Vis spectroscopy was used to detect the formed nanometals. Au-NPs synthesis was confirmed by the presence of surface plasmon resonance (SPR) of Au-NPs at 520–528 nm as measured by a UV-Visible spectrophotometer 1601 pc, Co., Ltd., Kyoto, Japan (Figure 1c). In addition, the cell-free supernatant of *Streptomycin* S91 provided stable biosynthesized Se-NPs with a distinctive orange color and a wavelength of maximum absorption peak in the UV-Vis spectra at 450 nm (Figure 1d).

#### 2.3.2. Determination of Particle Size and Zeta Potential

**Figure 2:**
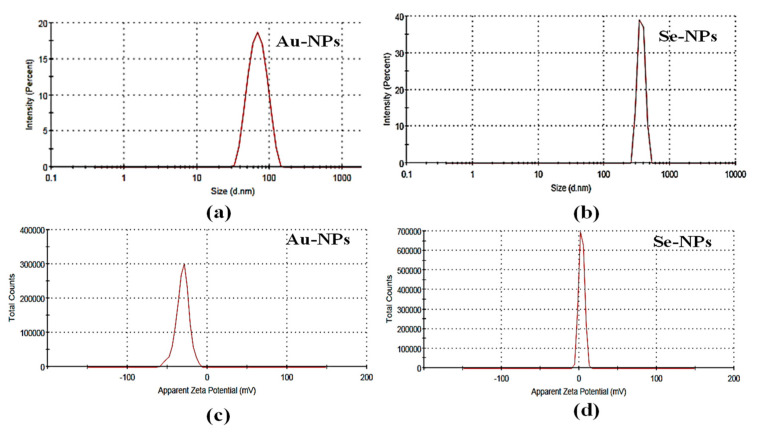
Particle size distribution of (**a**) Au-NPs and (**b**) Se-NPs and Zeta potential of (**c**) Au-NPs and (**d**) Se-NPs.

The particle size of the bioformed nanometals was detected by the dynamic light scattering (DLS) technique of laser light using a particle size analyzer (Microtrac, nanotrac wave II Q, MRB, VERDER, Haan, Germany). The tested samples were diluted and sonicated to eliminate any aggregations in the preparations. The Zeta potentials of the synthesized nanometals were determined using Microtrac, nanotrac wave II, Q to assess the electrophoretic mobilities of the formed nanometals. The diameter of Au-NPs, and Se-NPs was 70.59 and 315.4 nm, respectively (Figure 2a,b). The Zeta potentials for, Au-NPs and Se-NPs were −29.7 mV and 3.88, respectively (Figure 2c,d).

#### 2.3.3. TEM Imaging

**Figure 3:**
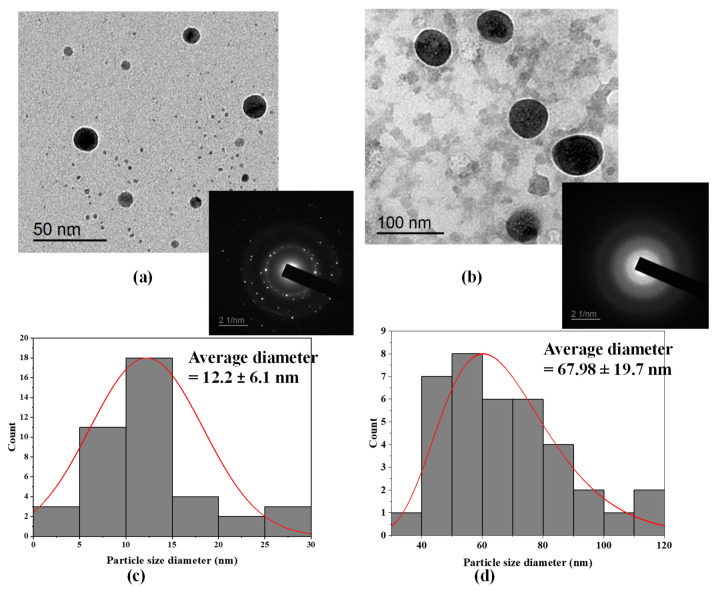
Transmission electron microscopic imaging along with SAED of the biosynthesized (**a**) Au-NPs and (**b**) Se-NPs, (**c**) Au-NPs particle size histogram and (**d**) Se-NPs particle size histogram.

The shape and size of the biosynthesized Au-NPs and Se-NPs using culture supernatants of S91 were elucidated by TEM imaging (Figure 3). TEM analysis elicited monodispersed Au-NPs with a spherical shape (Figure 3a). The average diameter of the synthesized Au-NPs was 12.2 ± 6.1 nm (Figure 3c). The TEM image foretold the synthesis of uniform, spherical-shaped Se-NPs using cell supernatant of *Streptomycin* S91 and the average size estimated was 67.98 ± 19.7 nm (Figure 3d). In addition, the coat surrounding the Se-NPs has been observed to be composed of bacterial biological materials, such as lipids or proteins. This coating is mainly composed of organic material surrounding the selenium core [29].

To further confirm the crystal makeup of the prepared nanospheres, selected area electron diffraction pattern (SAED) analysis was performed. The SAED pattern of a single spherical gold particle confirmed the single crystalline nature of the Au-NPs due to presence of bright, discrete circular spots (Figure 3a). In Figure 3b the SAED analysis of Se-NPs is amorphous due to the diffused ring patterns.

#### 2.3.4. Energy Dispersive X-ray Diffraction (EDX)

**Figure 4:**
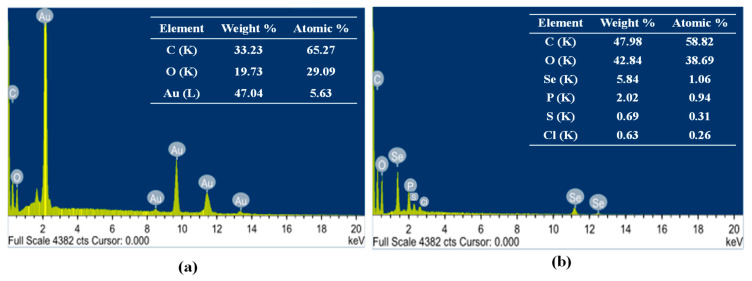
Energy dispersive X-ray diffraction (EDX) of the biosynthesized (**a**) Au-NPs and (**b**) Se-NPs.

The elemental composition of Au-NPs and Se-NPs was determined using energy dispersive X-ray diffraction (EDX). Strong signals of gold atoms were detected at various range of energy (2.2, 9.7 and 11.4 keV), indicating that gold is a major constituent in the nanostructures. However, other EDX peaks for C and O atoms were also detected (Figure 4a). EDX analysis of the prepared Se-NPs revealed the characteristic selenium absorption peaks at 1.37, 11.22 and 12.4 keV. Moreover, Se-NPs showed different elemental composition of C, O, P, S and Cl (Figure 4b).

#### 2.3.5. FTIR Spectroscopy Measurements

**Figure 5:**
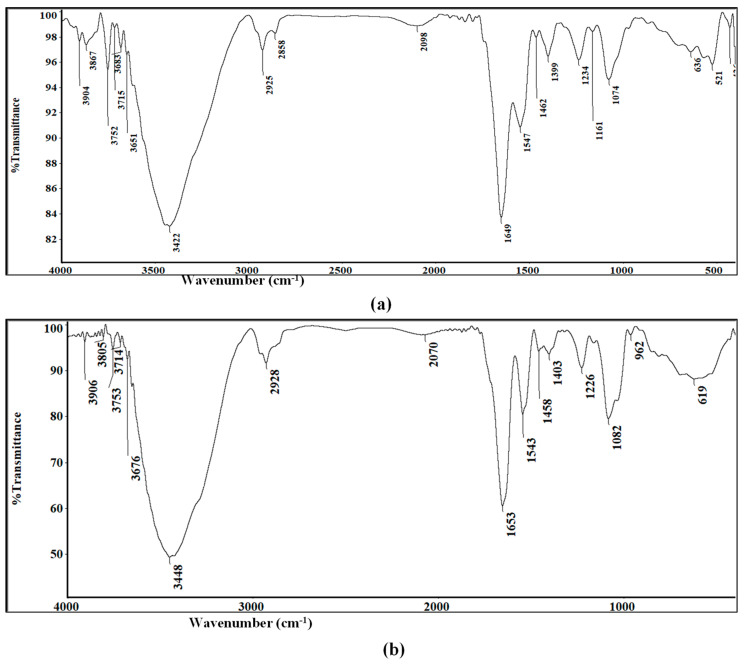
Fourier-transform infrared spectroscopy (FTIR) of (**a**) Au-NPs and (**b**) Se-NPs.

FTIR spectra were performed for further confirmation of nanometals biosynthesis. Analysis of Au-NPs presented many functional groups showing peaks at 3422, 2925, 2858, 1649, 1547, 1234, 1161, and 1074 (Figure 5a). Similarly, Se-NPs IR spectrum revealed strong absorption bands at 3448, 2928, 1653, 1543, 1226, and 1082 cm^−1^ (Figure 5b).

Broad and strong peaks at 3422 and 3448 cm^−1^ for Au-NPs and Se-NPs, respectively, corresponds to O–H stretch, H–bonded of alcohols and phenols involved in their reduction. These peaks also indicated the possibility of stretching vibrations (N–H) of primary and secondary amines. Bands at 2925 cm^−1^ for Au-NPs and 2928 cm^−1^ for Se-NPs correspond to aldehyde C–H stretching. FTIR spectrum of Au-NPs showed a low intensity band at 2858 cm^−1^ that indicated the presence of methylene group (CH2) next to OH or NH2 functional group. Sharp bands at 1649 and 1653 cm^−1^ for Au-NPs and Se-NPs were, respectively, assigned for (NH) C=O and C-C stretching indicating the presence of proteins. In addition, the amide II group of the proteins were proven through sharp peaks at 1547 cm^−1^ in Au-NPs and 1543 cm^−1^ in Se-NPs. The peaks at 1234, 1161, 1074 cm^−1^ for Au-NPs and 1226, 1082 cm^−1^ for Se-NPs were assigned to C–N stretched aromatic and aliphatic amines.

### 2.4. Genotypic and Morphological Characters of Streptomyces S91

**Figure 6:**
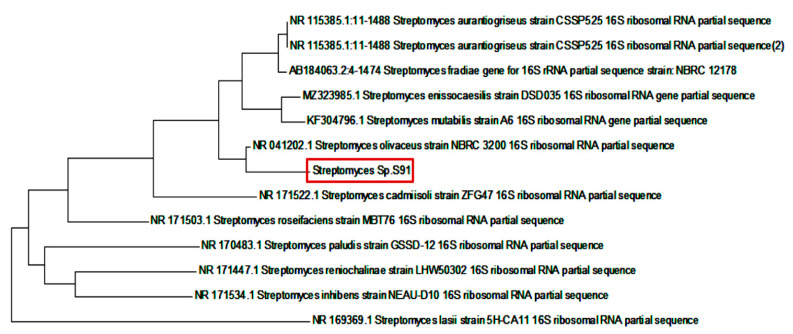
Phylogenic analysis of 16S rRNA of *Streptomyces* isolate S91 with high similarity to *Streptomyces olivaceous* strain NBRC 3200.

The partial sequence of the 16S rRNA gene of isolate S91 was submitted to GenBank (NCBI) under accession number OK427287. A phylogenetic tree was constructed using the neighbor-joining method (Figure 6). The resemblance of the 16S rRNA partial gene sequence was analyzed with the similar existing sequences available in the data bank of NCBI using a BLAST search (https://blast.ncbi.nlm.nih.gov/Blast.cgi, accessed on 8 October 2021). The DNA sequences were aligned and a dendrogram was constructed by the neighbor joining method [30]. Compared to NCBI GenBank sequences, *Streptomyces* S91 showed more similitude to *Streptomyces olivaceous* strain NBRC 3200 (98.82% sequence identity).

**Figure 7:**
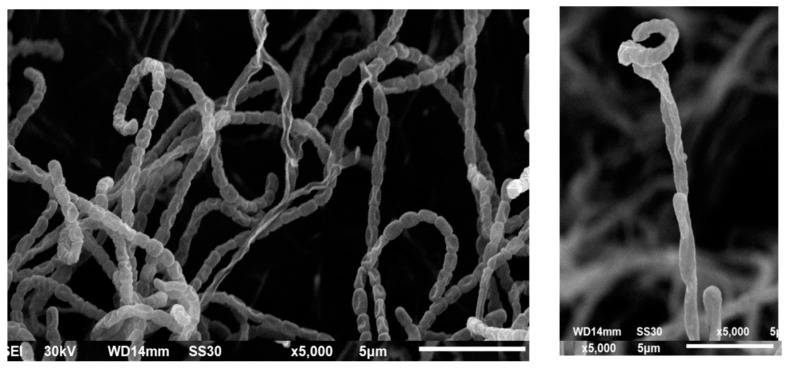
Scanning electron microscopic analysis of the spores of *Streptomyces* S91.

Scanning electron micrographs of *Streptomyces* S91 spores indicated that S91 has smooth, spiral spore chains with smooth surface, as indicated in Figure 7.

### 2.5. Anti-QS Activity of the Biosynthesized Nanometals

**Figure 8:**
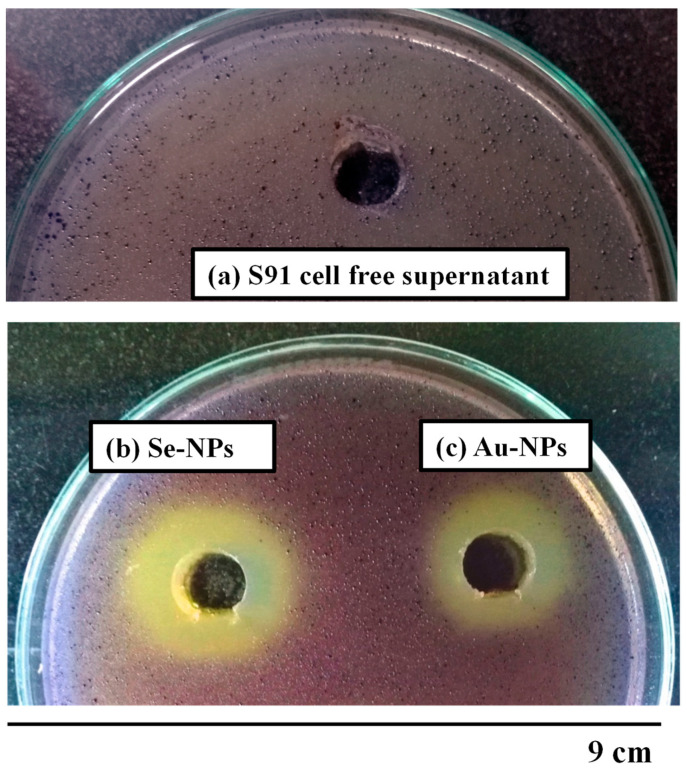
Screening for anti-QS activity using *C. violaceum* ATCC 12472 biosensor strain, showing zone of pigment inhibition of (**a**) the cell free supernatant, (**b**) Se-NPs, and (**c**) Au-NPs.

The reporter *C. violaceum* strain ATCC 12472 was used to assess the potential QSI effects of the synthesized nanometals. The cell free supernatant of *Streptomyces* S91 exhibited no change in the violet color of the reporter strain. Both Au-NPs and Se-NPs decreased the violacein production by 11- and 7.5-mm zone diameter of the turbid pigmentless halo, respectively (Supplementary Table S3, Figure 8).

### 2.6. Minimal Inhibitory and Minimal Bactericidal Concentration

A series of *P. aeruginosa* standard strains, PAO-JP2, PAO1, PAO14, and clinical isolates KU6 and 52 were selected. The minimum inhibitory concentrations (MICs) of nano-synthesized particles were assessed and compared with those obtained for ceftazidime (CAZ) and ciprofloxacin (CIP) (Table 1). The MICs of Au-NPs were in the range of 9.2–36.9 μg/mL and Se-NPs were in the range of 4.6–1184.4 μg/mL. In the same way, the MICs of CAZ and CIP ranged between <2–64 μg/mL and <2–256 μg/mL against all test pathogens, respectively. Sub-MICs of each nano-synthesized particle (1/2 and 1/4 MICs) were also assigned (Table 1).

### 2.7. Effect on Bacterial Viability

**Figure 9:**
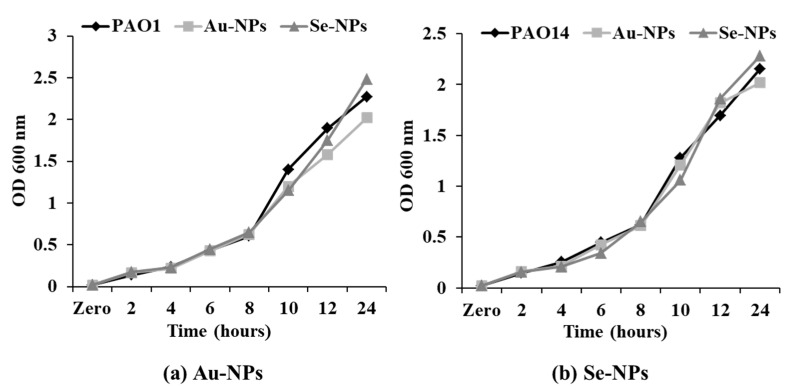
Growth curve analysis of (**a**) PAO1 and (**b**) PAO14 in the presence of 1/2 MIC of Au-NPs and Se-NPs every 2 h time interval measured at OD 600 nm. Data represent the means of triplicate independent experiments.

The growth pattern of *P. aeruginosa* PAO1 and PAO14 was not significantly affected upon treatment with sub-MICs of Au-NPs and Se-NPs (Figure 9). Thus, concentration levels below MICs (1/2 and 1/4 MICs) have further been used to determine the inhibitory properties of NPs on QS-related virulence factors and biofilms formation.

### 2.8. Anti-Biofilm Effect of the Prepared Nanometals

**Figure 10:**
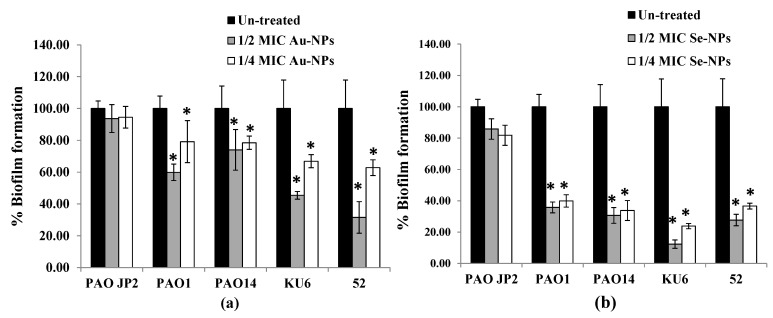
Effect of sub-inhibitory concentrations (1/2 and 1/4 MICs) of (**a**) Au-NPs and (**b**) Se-NPs on biofilm formation by *P. aeruginosa* PAO-JP2, PAO1, and PAO14 standard strains and KU6, and 52 clinical isolates. The data are presented as percentage biofilm formation (*, significant, *p* < 0.05). Error bars represent the SD of three independent experiments.

The anti-biofilm property of Au-NPs and Se-NPs, at respective sub-MICs, against *P. aeruginosa* tested isolates was determined by crystal violet (CV) method. The sub-MIC levels of nano-biosynthesized particles, when incubated with *P. aeruginosa* cells culture overnight, exhibited concentration-dependent biofilm inhibition (Figure 10, Supplementary Figure S1). In comparison to a dense biofilm of control untreated isolates, Au-NPs at 1/2 and 1/4 MICs reduced biofilm formation by 26–68% and 21–37%, respectively (Figure 10a). Se-NPs exhibited a 64–88% and 63–76% reduction in biofilm biomass at 1/2 and 1/4 MICs, respectively (Figure 10b).

### 2.9. Virulence Factors Inhibition by the Formed Nanometals

**Figure 11:**
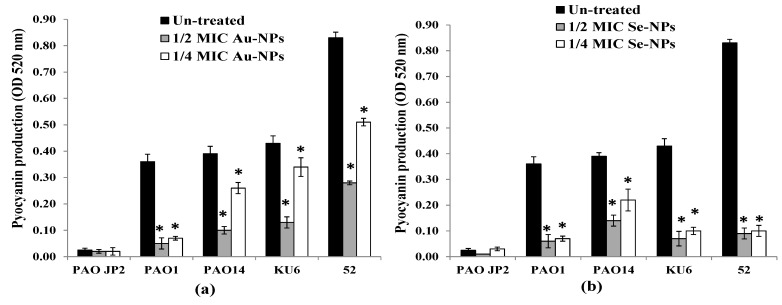
Impact of sub-inhibitory concentrations (1/2 and 1/4 MICs) of (**a**) Au-NPs and (**b**) Se-NPs on pyocyanin production by *P. aeruginosa* PAO-JP2, PAO1, and PAO14 standard strains and, KU6, and 52 clinical isolates compared with the untreated cultures (*, significant, *p* < 0.05). Error bars represent SD of three independent experiments.

Production of pyocyanin from *P. aeruginosa* PAO-JP2, PAO1, PAO14 standard strains, and KU6, 52 clinical isolates in the presence and absence of Au-NPs and Se-NPs sub-MICs was determined. The findings presented in Figure 11a revealed that 1/2 and 1/4 MICs of Au-NPs caused a significant loss of pyocyanin pigment production by 40–88% and 20–82%, respectively (*p* < 0.05) compared to untreated isolates. Pyocyanin inhibition was verified using Se-NPs by 65–90% and by 43–88% with 1/2 and 1/4 MIC, respectively (*p* < 0.05, Figure 11b).

**Figure 12:**
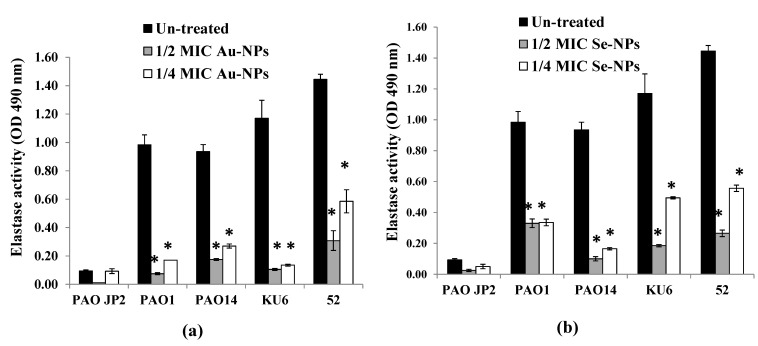
Effect of sub-inhibitory concentrations (1/2 and 1/4 MICs) of (**a**) Au-NPs and (**b**) Se-NPs on elastase production by *P. aeruginosa* PAO-JP2, PAO1, and PAO14 standard strains and, KU6, and 52 clinical isolates compared with the untreated cultures (*, significant, *p* < 0.05). Error bars represent SD of three independent experiments.

The effect of sub-MICs of Au-NPs and Se-NPs on the production of elastase enzyme was also investigated by the elastin Congo red (ECR) technique. Compared to the control, Au-NPs and Se-NPs eliminated the elastase activity by 79–92% and 60–89% (*p* < 0.05), respectively in concentration-dependent manner (Figure 12).

**Figure 13:**
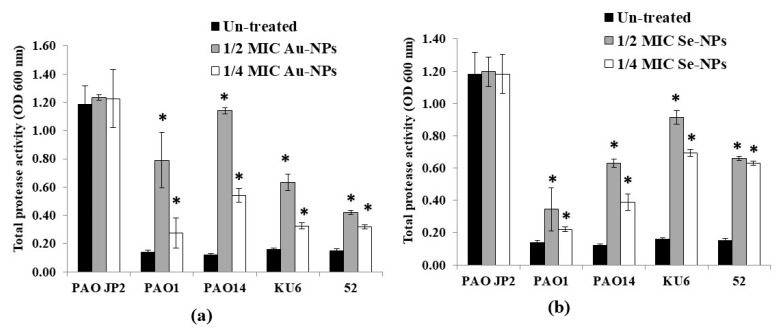
Effect of sub-inhibitory concentrations (1/2 and 1/4 MICs) of (**a**) Au-NPs and (**b**) Se-NPs on protease activity of *P. aeruginosa* PAO-JP2, PAO1, PAO14 standard strains and KU6, 52 clinical isolates compared with the untreated cultures (*, significant, *p* < 0.05). Error bars represent SD of three independent experiments.

Furthermore, the production of protease enzymes in the presence of sub-MICs of biologically prepared NPs was assayed using the skimmed milk method. As shown in Figure 13, the increase in OD600 nm reading, indicated the lowest proteolytic activity of the compound. At both 1/2 and 1/4 MICs, Au-NPs caused a significant reduction (*p* < 0.05) in the total protease activity, up to 89% and 78% compared to untreated *P. aeruginosa* isolates, respectively. While the maximum protease reduction in the tested isolates upon treatment with 1/2 and 1/4 MICs of Se-NPs reached 83% and 77%, respectively, as compared to the plain non-treated isolates.

### 2.10. RT-PCR Analysis

**Figure 14:**
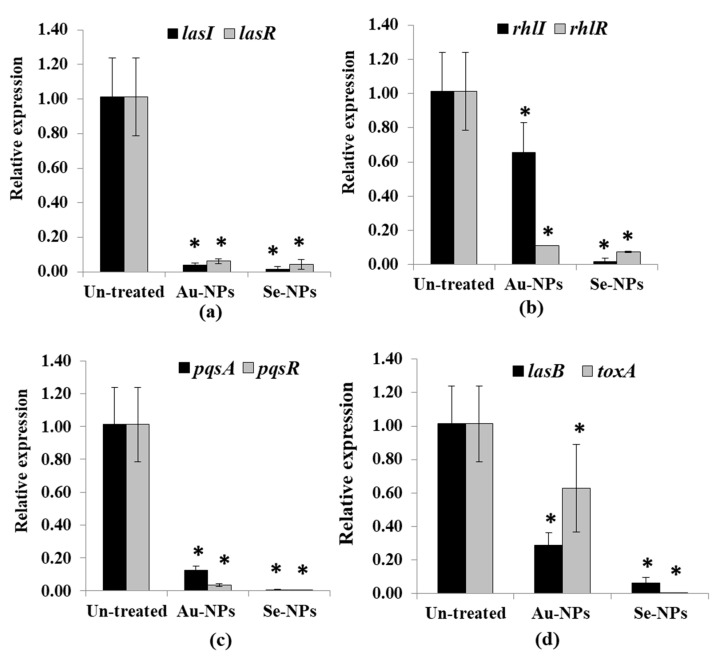
Significant reduction of the QS cascade in *P. aeruginosa* PAO1 treated with Au-NPs and Se-NPs (1/2 MIC). Relative expression of (**a**) *lasI* and *lasR*, (**b**) *rhlI* and *rhlR*, (**c**) *pqsA* and *pqsR* and (**d**) *lasB* and *toxA* compared with the untreated cells. Error bars indicate SD of independent three experiments (* significant *p* < 0.05).

The expression of QS-regulated genes in both treated and untreated PAO1 was assessed using RT-PCR. The relative expression of QS genes *lasI*, *lasR*, *rhlI*, *rhlR*, *pqsA*, and *pqsR* was calculated from Ct values for each isolate and normalized to its average relative amount of standard *rpoD* gene. Treatment with 1/2 MIC of Au-NPs and Se-NPs significantly suppressed *lasI* expression in PAO1 by 97% and 95% and *lasR* genes by 99% and 97%, respectively (*p* < 0.05, Figure 14a). Moreover, a significant decrease in the expression of *rhlI* and *rhlR* was observed as 36% and 90% for Au-NPs, and 99%, and 94% for Se-NPs-treated PAO1 (*p* < 0.05, Figure 14b). Furthermore, Au and Se-NPs significantly reduced the relative expression of *pqsA* in PAO1 by 89% and 99.9% and lowered *pqsR* relative expression by 98% and 99.9%, respectively (*p* < 0.05, Figure 14c).

Additionally, Au-NPs and Se-NPs caused a significant reduction in the expression level of *lasB* and *toxA* in the range of 38–99.9%, relative to untreated PAO1 (*p* < 0.05, Figure 14d).

## 3. Discussion

Increasing antimicrobial resistance is a global health crisis. It was thought that the war with microorganisms was gained. Microorganisms have the ability to develop resistance to new antimicrobials through different mechanisms, either natively to the organisms or acquired from another one [3].

Nanometals and nano-oxides have been identified for their antimicrobial effects [4] such as silver [31], selenium [21], gold [32], aluminum [33], aluminum oxide [34], zinc oxide [35], copper oxide [36], and iron oxide [37]. Different physical and chemical methods are the primary ways to synthesize nanometals. However, disadvantages such as the use of costly precursors, with the production of hazardous wastes, in addition to toxicity, high reactivity, and instability, have been developed. Therefore, there is an increasing need for the biological synthesis of nanometals [38]. Biosynthesized nanometals such as Ag-NPs and Au-NPs exhibit safety and activity for medical applications [39,40,41]. Au-NPs synthesized from *Lignosus rhinocerotis* sclerotia extract and chitosan inhibited many foodborne pathogens such as *Bacillus*, *E. coli*, *P. aeruginosa*, and *S. aureus* [42]. Bacteria are excellent tools for nanometal fabrication due to their diversity and adaptability to extreme environmental conditions [43]. For example, *P. aeruginosa* was found to synthesize Au-NPs [44] and *Klebsiella pneumoniae* was also reported for Se-NPs biosynthesis [45]. However, utilization of *Streptomyces* for green synthesis of nanometals has received much less attention and could provide a successful tool in the preparation of biocapped nanometals for combating microbial pathogenies and microbial virulence.

In this study, the cell free supernatant of *Streptomyces* isolate S91 synthesized Au-NPs and Se-NPs within 48 h. However, the synthesis of Se-NPs by other *Streptomyces* isolates was delayed. Therefore, isolate S91 was selected for the synthesis of nanoscale Au-NPs and Se-NPs (Supplementary Table S2).

The cell-free supernatant of *Streptomyces* S91 turned dark purple after exposure to HAuCl_4_, as an indication of Au-NPs synthesis (Figure 1a), while turned orange color with the biosynthesized Se-NPs (Figure 1b). Nanometals exhibited peculiar optical absorption spectra in the UV-Vis region due to surface plasmon resonance. The surface plasmon resonance indicates the specific vibration mode of electrons on the surface of nanometals, which differs according to the shape and size of the nanometals.

The synthesis of Au-NPs increased with time, reaching its maximum after 72 h. Au-NPs exhibited a maximum peak at 520–528 nm after 48 h due to collective oscillation of the electron band conduction around the nanoparticle’s surface (Figure 1c). Similarly, fabrication of biogenic Au-NPs has been attained at a maximum absorption peak at 520–550 nm by *bacillus niabensis*, *Streptomyces griseoruber*, and *Streptomyces microflavus* [46,47,48]. Additionally, Au-NPs produced by *Streptomyces* sp. isolated from acid forest soil have maximum spectra of 564 nm [49].

Se-NPs biosynthesis began after 48 h of incubating SeO_2_ with the supernatant of S91. The orange coloration indicated the formation of Se-NPs. Nano Se showed a maximum peak at 450 nm associated with free electrons and excitation of longitudinal plasmon vibrations (Figure 1d). On the other hand, the bioproduction of Se-NPs by *Streptomyces griseoruber* was confirmed by UV-Visible spectrophotometer at 575 nm [46] and selenium nanorods produced by *Streptomyces bikiniensis* revealed a sharp peak at 620 nm [50]. The variation in the spectrum mode for Se-NPs has been recognized with variation in the morphological characters, various shapes, size, and aggregates of the biosynthesized Se-NPs. The results of the particle size distribution pattern and Zeta potential of the prepared Se-NPs, and Au-NPs were determined by the light scattering method (Figure 2), with a polydispersity index of 0.1–0.219, indicating a homogenous size distribution. A high Zeta potential value indicates the colloidal stability of the prepared nanometals. The electrostatic repulsive forces between the particles hinder the prepared nanometals from accumulation or clumping.

TEM images elucidated that the average diameter of the synthesize Au-NPs was 12.2 ± 6.1 nm (Figure 3a,d). Similarly, Au-NPs produced by the extracellular secretion of *Bacillus niabensis* exhibited a particle size in the range of 10–20 nm [48]. For instance, the biosynthesis of Au-NPs by *Streptomyces viridogens strain HM10* isolated from the Himalayan Mountains by Balagurunathan and colleagues possessed an average particle size of 18–20 nm [51]. *Marinobacter pelagius*, a submarine isolate, synthesized Au-NPs with polymorphic shapes spherical and triangles [52]. Intercellular synthesis of Au-NPs by thermophilic filamentous fungi [53] provides nanometals with different shapes; spherical and hexagonal Au-NPs and different sizes between 6–40 nm. In addition, Au-NPs constructed with *Streptomyces griseoruber* have a spherical shape and a particle size range of 5–50 nm [47].

In addition, the average size of the synthesized Se-NPs was 67.98 ± 19.7 nm (Figure 3c,d). Some studies have reported the potential of *Streptomyces* to synthesize Se-NPs. The biosynthesis of nanometals involves two steps, the reduction of the metal ions by diverse metabolites, amino acids, monosaccharides, and cofactors, followed by capping the formed NPs with proteins that stabilize the formed nanometal. At the isoelectric point, proteins can attach to the surface of nanometals by Van der Waals forces, forming bonds with nitrogen and sulfur atoms of the proteins and metal ions that enhance metal stabilization [54].

It was also noticed that the average size of NPs determined by TEM was smaller than that determined by the DLS technique. The particle size estimated by TEM represents the exact size of the prepared nanometals as detected in the dry form, while the diameter obtained by the DLS technique represents the hydrodynamic size of the particles in the hydrated state. Hence, the particles will show a larger hydrodynamic size due to the solvent effect in the hydrated state [55]. Herein, SAED is used to efficiently elucidate the crystallinity of Au-NPs and Se-NPs [56]. SAED data revealed well-crystallized single crystals of Au-NPs [57] and the amorphous or non-crystalline nature of the synthesized Se-NPs (Figure 3a,b) [58,59].

The EDX microanalysis was performed to identify the composition of biogenic nanometals. Au-NPs exhibited a higher weight percentage of gold, 47.04%. Among the major peaks in the Au-NPs spectrum, the peak appearing at 2.2 keV is a characteristic peak of gold (Figure 4a). The presence of other EDX peaks for O and C is logical as they came from *Streptomyces* supernatant macromolecules which bound to Au-NPs surface [60]. The elemental composition of Au-NPs has also been analyzed previously using EDX with similar peak profile [61,62]. In addition, the elemental content of Se-NPs was confirmed by the presence of a peak at 1.37 keV in EDX (Figure 4b), which is the characteristic peak for SeLα [63,64]. On the other hand, EDX spectrum of Se-NPs showed a selenium percentage in weight of only 5.84%. This low percentage came in accordance with Cremonini and colleagues who provided very low selenium weight in biogenic nanometals [65]. Similarly, the composition of biogenic Se-NPs was rich in C, O, P, S and Cl. Besides, the high carbon weight (47.98%) suggests that the organic shell layer on the surface of Se-NPs may be a protein from *Streptomyces* cells, acting as a capping agent during particle formation [66].

FTIR was also used to recognize the functional groups of each component present in each type of biologically synthesized nanoparticle that are involved in the bioproduction of Au-NPs and Se-NPs (Figure 5). The extracellular secretion of protein in the *actinomycetes* filtrate act as capping agent over the surface of nanometals, hence responsible for bioreduction, stabilization and inhibit aggregation of nanometals [67,68].

In this study, *Streptomyces* S91 could tolerate high salt concentrations. This high salt adaptation might be acquired due to mutation during the habitat in the microenvironment. Therefore, *Streptomyces* S91 could produce new metabolites associated with the synthesis of nanomaterials [69]. The 16S rRNA and phylogenetic analysis revealed that *Streptomyces* isolate S91 had a high similarity (98.82%) to *Streptomyes olivaceus* (Figure 6). Recently, a relationship between taxonomy and secondary metabolite production, especially for the *Streptomyces* genus was investigated. Based on the genetic analysis and phylogenetic features, *Streptomyces* S91 could be considered a novel species of *S. olivaceous* to produce Au-NPs and Se-NPs from an aqueous solution containing HAuCl_4_ and SeO_2_ salts, respectively.

*P. aeruginosa* is one of the most dominant pulmonary pathogens, causing a high rate of morbidity and mortality in cystic fibrosis patients [46]. It utilizes QS, bacterial crosstalk, which is a director for virulence factors production, to be victorious over human health. Herein, the effect of Au-NPs and Se-NPs, biologically synthesized from S91 cell-free supernatant, on QSI and biofilm assembly was demonstrated.

*C. violaceum* ATCC 12472 was used for assessment of the QSI inhibitory effect of Au-NPs and Se-NPs by observing non-pigmented zone, with turbid growth surrounding the tested nanometals (Table S3, Figure 8). *C. violaceum* ATCC 12472 is a violacein-producing bacteria, used to screen the anti-QS potential of the tested compounds [47,48]. Violacein pigment production is driven by QS signaling molecules, so the elimination of violacein purple color without bacterial growth inhibition indicates the interaction with acyl homoserine lactone (AHL) regulated QS and subsequently, QS interruption [49,50]. Then, the growth pattern of *P. aeruginosa* standard strains PAO1 and PAO14 at 1/2 MICs of Au-NPs and Se-NPs was the same as the growth pattern of the untreated cultures (Figure 9).

In the next steps, the antivirulent effects of Au-NPs and Se-NPs were detected at sub- MICs which confirms that the significant reduction in different virulence factors and in the expression of QS genes is not due to the antimicrobial activity of the synthesized Au-NPs and Se-NPs. Singh and coauthors also reported QS inhibition in PAO1 when AgNPs were used at sub-MICs [51].

Biofilm-related *P. aeruginosa* infections are becoming difficult to solve [5], due to the greater drug resistance of biofilm-resided cells than that of their planktonic counterparts [70]. QS is a maestro for bacterial biofilm construction, virulence activation, and antibiotic resistance [71], so interruption of biofilm is achieved by QS deactivation. Disaggregation of *P. aeruginosa* biofilm and virulence factors by the resultant NPs were identified at sub-MIC levels. Both Au-NPs and Se-NPs significantly reduced the biofilm over the same concentration range, compared to untreated samples (Figure 10). Previous research has shown that Au-NPs [72], Fucoidan-stabilized Au-NPs [62], silica NPs [73], and Se-NPs synthesized by Gram-negative bacteria [65] inhibit *P. aeruginosa* biofilm architecture.

*P. aeruginosa* pathogenicity is managed by the production of a spacious virulence armory [74]. Among these armories, pyocyanin, elastase, and protease were chosen to examine their production under sub-MIC levels of Au-NPs and Se-NPs. Elastase and total protease enzymes support systemic dissemination of *P. aeruginosa* as well as host tissue and immunity degradation, while the green-pigmented pyocyanin causes oxidative and cytotoxic damage [75]. We proved that the biogenic Au-NPs and Se-NPs significantly reduced the levels of pyocyanin, total protease, and elastase, without any effect on bacterial growth or initiating drug resistance. Both Se-NPs and Au-NPs inhibited pyocyanin production in range of 43–90% and 20–88%, respectively (Figure 11). On the other hand, the reduction in the elastolytic and proteolytic activity of Au-NPs (up to 92% and 89%, respectively) was higher than that of Se-NPs (up to 89% and 83%, respectively) (Figure 12 and Figure 13). In the study of Prateeksha et al., a decrease in elastase and protease production was observed, after treatment with selenium nanovectors at sub-MIC [76]. The reduction of pyocyanin, elastase, protease, and biofilm formation by Au-NPs and Se-NPs explains their anti-QS activity. This conforms to various reports on the inhibition of virulence and biofilm production by anti-QS nanostructured materials in *P. aeruginosa* [76,77]. Interruption of QS, therefore, provides a potential anti-virulent therapy to be used in the future [78].

QS in *P*. *aeruginosa* is a convoluted system, composed of two common cascades, *lasIR* and *rhlIR* in addition to its specific PQS system. QS-regulated genes are mainly under the control of the *las* and/or *rhl* QS cascade. The PQS system acts as a link between both QS systems, *las* and *rhl*, and can regulate the expression of several virulence factors in *P. aeruginosa*, such as pyocyanin development [8,79].

Treatment with Au-NPs and Se-NPs induced inhibition of virulence factors (pyocyanin, elastase, and protease) and biofilm formation, but these effects were not found in the *lasR* mutant [76]. At the molecular level, Au-NPs and Se-NPs banned *lasR* from doing their job as transcriptional activators through significant reductions in *lasR* expression by 95% and 97%, respectively (Figure 14a). Furthermore, the expression of *lasI*, *rhlI*, *rhlR*, *pqsA*, *pqsR*, *lasB* and *toxA* was downregulated upon Au-NPs and Se-NPs treatment (Figure 14). As *las* being the prominent QS system in *P*. *aeruginosa*, controlling other *rhl* and *pqs* circuits [80,81]. Our results support that inhibition of *P*. *aeruginosa* QS signaling has occurred via *lasR* down expression. As a result of the QS interruption, virulence factors were inhibited, and *P. aeruginosa* infection was delayed.

## 4. Materials and Methods

### 4.1. Microorganisms, Media, and Chemicals

Reporter strain *C. violaceum* ATCC 12472 was kindly provided by Prof. Dr., McLean (Department of Biology, Texas State University, San Marcos, TX, USA). *C. violaceum* ATCC 12472 was grown on Luria-Bertani (LB) broth (tryptone 10 gm, yeast extract 5 gm, sodium chloride 10 gm, distilled water to 1000 mL; pH 7.2) and solidified using bacteriological agar 20 gm/L. *Streptomyces* isolates were isolated, purified, and propagated on ISP2 media (malt extract 10 gm, yeast extract 4 gm, glucose 4 gm, agar 20 gm; pH 7.2). All media used were purchased from Oxoid (Oxoid Limited, Basingstoke Hants, UK). All the chemicals used, selenium oxide (SeO_2_) and chloroauric acid (HAuCl_4_), were obtained from Sigma Aldrich, UK with high purity. *P*. *aeruginosa* standard strains PAO1 and PAO14 were used as positive controls. *P. aeruginosa* clinical isolates kU6 and 52 were purified from urine samples. *P. aeruginosa* QS double-mutant strain, PAO-JP2 (Δ*lasI::Tn10, Tcr;* Δ*rhlI::Tn501-2,Hgr*) was used as a negative control [82]. The strains were retained as frozen glycerol stocks at −80 °C.

### 4.2. Isolation of Soil Microorganisms

Different soil and submarine samples (20 mg) were collected from an area 20 cm below the soil surface in a clean container according to Jeffrey (2008) [83]. Soil samples were dried in an oven at 50 °C for 10 min. One gram of the collected soil sample was suspended in 10 mL of sterile NaCl (0.85%). Each sample was mixed for 20 min. The prepared soil suspension was diluted at 1:10 in a sterile saline solution with good mixing. The diluted soil suspensions 10^8^ and 10^7^ were platted onto ISP2 plates.

### 4.3. Purification, Microscopic Examination, and Storage of Bacterial Isolates

The plates were morphologically examined for the characteristic shape of *Streptomyces*, with rough and chalky colonies. Various *Streptomyces* species are black, gray, and white. *Streptomyces* colonies were selected and scraped onto ISP2 transfer media and the plates were incubated for 7 days at 28 °C [83]. A single colony of each isolate was separated again on sterile ISP2 plates. The isolated colonies were stained with Gram stain and examined under the microscope.

### 4.4. Screening Streptomyces Isolates for Survival under High Salt Concentrations

Pure *Streptomyces* colonies were transferred to fresh media, ISP2 media, supplemented with different concentrations of metal salts, and a lawn of *Streptomyces* culture was grown at 30 °C for 7 days. The isolates that survived in high salt concentrations were collected and stored on ISP2 slants and were selected for the synthesis of nanometals [84].

### 4.5. Synthesis of Nanometals

The selected *Streptomyces* isolates were inoculated on ISP2 agar media. Spores were collected by surface scraping in tween water (0.1 *v*/*w*). The biosynthesis of nanometals was carried out via inoculating ISP2 media (50 mL) with the prepared spores of *Streptomyces* (1/10^6^ CFU/mL) and cultivating them in a rotary shaker at 30 °C, 200 rpm for 5–7 days. Cultures were removed by centrifugation at 6000× *g* for 15 min at 4 °C and used to make nanometals. The cell-free supernatant (pH, 5.3) was collected and combined with 60 mM selenium oxide (SeO_2_) and 3 mM of chloroauric acid (HAuCl_4_) for the synthesis of Se-NPs and Au-NPs, respectively. A control flask composed of culture supernatant without SeO_2_ and HAuCl_4_ was also included. In addition, ISP2 medium mixed with the same concentration of the used salts, SeO_2_ and HAuCl_4_, were also included in each run. Samples of the reaction solution were collected at 24 and 48 h and used in the detection and characterization of the biologically formed nanometals [85]. The ability of the selected *Streptomyces* isolates to synthesize different types of nanometals was confirmed by spectroscopy analysis using a UV-Vis spectrophotometer 1601 pc at room temperature and a resolution of 1 nm at a wavelength range of 300–1000 nm. The cell-free supernatants, which were collected at 24, and 48 h and treated with different metals, were analyzed by UV-Vis spectrum with detection of λ_max_. Culture supernatant was utilized as blank for each nano-preparation.

### 4.6. Characterization of the Synthesized Nanometals

#### 4.6.1. Color Change and UV-Visible Spectroscopic Analysis

The microbial reduction of metallic ions added to *Streptomyces* supernatant was initially monitored by observing the color change of the cell-free supernatants, which represents a primary indication of the reduction of metal ions and the formation of nanometals. In addition, the absorption spectra of the synthesized nanometals were detected in the wavelength range of 300–1000 nm using a UV-Vis spectrophotometer 1601 pc, Co., Ltd., Kyoto, Japan [86].

#### 4.6.2. Particle Size Analysis, Polydispersity Index, and Zeta Potential

The particle size, polydispersity index, and Zeta potential of the biosynthesized nanometals were determined by the DLS technique of laser light using the particle size analyzer Microtrac, nanotrac wave II Q, Microtrac, MRB, VERDER, Haan, Germany [86].

#### 4.6.3. Transmission Electron Microscopy

TEM imaging was used for detection of the morphological shape, and the dispersion of the formed nanometals (200 K TEM, JEOL JEM-2100, Pleasanton, CA, USA). TEM examination was performed by adding one drop of cell-free supernatant treated with different salts, SeO_2_ and HAuCl_4_, to a carbon-coated copper grid. Then, the excess unabsorbed solution was removed by a paper tissue. After dryness, the grid was assessed using TEM with a magnification power of X 1.000–800.000 (JEOL JEM-2100, Pleasanton, CA, USA). In addition, SAED of Au-NPs and Se-NPs was also observed with the same sample prepared for TEM analysis by selecting the specific area from the TEM images.

#### 4.6.4. Energy Dispersive X-ray Diffraction Analysis

A scanning electron microscope (SEM) (JEOL JSM 6510 lv, JEOL, Pleasanton, CA, USA) equipped with an EDAX micro-analytical system was used to determine the elemental composition of the analyzed nanometals. Thin films of nanometals were formed on glass cover slips by spreading the nanometals, and the samples were then coated with gold-palladium membranes and observed using a Jeol JSM-6510 L.V SEM (Pleasanton, CA, USA) at an accelerating voltage of 30 KV.

#### 4.6.5. FTIR Spectroscopy Measurements:

For FTIR spectral analysis, Au-NPs and Se-NPs solutions were centrifuged at 10,000 rpm for 15 min. The pellet was washed to remove the excess supernatant, followed by re-dispersion in deionized water. The sample was lyophilized, and the pure powdered forms of Au-NPs and Se-NPs were mixed with potassium bromide (KBr) to obtain pellets which were further analyzed using Thermo-Nicolet 6700 FTIR spectrophotometer (Thermo Scientific, Waltham, MA, USA) provided with ATR and with full integration with the OMNIC software. The FTIR spectrum was scanned in the range from 400 to 4000 cm^−1^ at a resolution of 4 cm^−1^. The peaks were plotted as the wavelength (cm^−1^) on the *y*-axis and percentage transmittance on the *x*-axis.

### 4.7. Characterization of Streptomyces 

#### 4.7.1. Molecular Characterization of Streptomyces Isolates

Isolates with high productivity of nanometals were identified by amplification and sequencing of the 16S rRNA gene. Spore suspensions of the tested *Streptomyces* isolates were prepared in sterile water. The spores were boiled for 10 min at 95 °C. The spore suspension was centrifuged at 6000 rpm and the cell lysate was transferred to another tube and kept at −20 °C [87]. PCR reaction mixture was performed using Phusion High-Fidelity PCR Kit (Thermo Scientific, Waltham, MA, USA) according to the manufacturers’ instructions and primer pairs 16S rRNA for (AGAGTTTGATCCTGGCTCAG) and 16S rRNA rev (AGAAAGGAGGTGATCCAGCC). As a negative PCR control, the reaction of PCR master mix without DNA temple was also performed. The conditions of PCR included an initial denaturation cycle at 95 °C for 2 min, repeated 35 cycles at 95 °C for 30 s, 47 °C for 30 s, and 72 °C for 30 s, and a final extension cycle at 72 °C for 10 min. The resulting PCR products were purified and sequenced [88,89].

#### 4.7.2. Morphological and Biochemical Characterization of Streptomyces Isolates

The morphological and cultural features of the used *Streptomyces* isolate were examined. The morphological characters include color and shape of the spore, surface characters of aerial mycelium, and substrate mycelium were detected. Additionally, diffusible, and melanoid pigments were perceived. Furthermore, the morphological characters of *Streptomyces* spores and their spore chains were examined by SEM (JEOL JSM 6510 lv, Pleasanton, CA, USA).

### 4.8. Antimicrobial and Antivirulence Effects of the Synthesized Nanometals

#### 4.8.1. Violacein Inhibition Assay by the Double Layer Agar Diffusion Method

The anti-QS potential of biologically synthesized nanometals was assessed using a reporter strain, *C. violaceum* ATCC 12472 by applying the double layer agar diffusion method. First, LB plates were poured and left to solidify. Then, *C. violaceum* culture (100 µL) was inoculated into LB soft media containing 0.7% *w*/*v* agar and poured on the surface of LB agar media. The cups were cut into the solidified agar plates. Approximately, 100 µL of the biosynthesized Au-NPs and Se-NPs were loaded into the wells. The plates were incubated at 28–30 °C for 48–72 h to examine the inhibition of purple violacein pigment development around each well, which is indicative of the QSI of the tested NPs [90,91].

#### 4.8.2. Evaluation of Minimum Inhibitory Concentration and Minimum Bactericidal Concentration

MICs/MBCs are the lowest concentrations of a chemical or drug that completely inhibits or kills bacteria. MICs and MBCs of Au-NPs and Se-NPs were calculated against *P. aeruginosa* PAO-JP2, PAO1, and PAO14 standard strains, and KU6, and 52 clinical isolates according to Clinical and Laboratory Standards Institute standards [92]. The MIC was performed using a micro-broth dilution standard, while the MBC was performed on the MHA plates. For the MIC, a 2-fold serial dilution of the biosynthesized nanometals was prepared in a 96-well microtiter plate. The diluted bacterial inoculums were added to each well, giving a final concentration of 1 × 10^5^ CFU/mL. The MICs of CAZ, CIP, and *Streptomyces* S91 supernatant were also included. All plates, including positive and negative controls and were incubated at 37 °C for 24 h to detect MICs. Concentrations below the MICs (1/2 MIC and 1/4 MIC) were considered the sub-inhibitory concentrations and were further used to study the inhibitory effect of nanometals on biofilm and QS-related virulence. The MBCs values were estimated by plating the suspension from each clear well of microtiter plates onto MHA plate and incubating them overnight at 37 °C to detect the MBC value.

#### 4.8.3. Effect of the Biosynthesized Nanometals on the Viability of *P. aeruginosa* Isolates

The growth profile of *P. aeruginosa* PAO1 and PAO14 in the presence of 1/2 MICs of Au-NPs and Se-NPs was checked by measuring the OD600 nm at 2 h time intervals up to 24 h during incubation under agitation (100 rpm) [93]. The growth of untreated *P. aeruginosa* PAO1 and PAO14 cultures was detected under the same conditions.

#### 4.8.4. Biofilm Inhibition Assay

In this study, quantitative detection of biofilm formation in the presence and absence of the prepared nanometals was estimated in 96-well flat-bottom polystyrene microtitre plates using the CV method [94]. All reference and clinical *P. aeruginosa* isolates were diluted to 0.5 McFarland and then challenged with sub-MICs (1/2 and 1/4 MICs) of Au-NPs and Se-NPs. After overnight incubation at 37 °C, the planktonic cultures were rejected, and the resultant biofilm was washed twice and fixed with 200 µL of methanol for 15 min. After removal of excess methanol, plates were dried and 200 µL of 1% *w*/*v* CV was used for staining planktonic cells for 15 min. Excess dye was removed, and the biofilms were quantified by solubilizing the adhered CV dye in 33% (*v*/*v*) glacial acetic acid (200 µL). The absorbance of the plate was monitored at 570 nm using a microtiter plate reader and the obtained data were analyzed to calculate the percentage inhibition of biofilm formation by Au-NPs and Se-NPs compared with untreated cultures.

#### 4.8.5. Quantitative Estimation of Virulence Factors

The impact of sub-MICs (1/2 and 1/4 MICs) of biologically synthesized Au-NPs and Se-NPs on the production of *P. aeruginosa* virulence factors, such as pyocyanin, elastase, and total protease, was assayed in the present study. All measurements were performed in triplicate.

##### Pyocyanin Level Assay

Pyocyanin pigment production of *P. aeruginosa* standard strains PAO-JP2, PAO1, and PAO14 in addition to clinical isolates KU6 and 52, untreated and treated with sub-MICs (1/2 and 1/4 MIC) of Au-NPs and Se-NPs was quantified according to the previously reported assay [95]. Briefly, 5 mL of the tested bacterial cultures were centrifuged, and the pyocyanin-contained supernatant was extracted with 3 mL of chloroform and then re-extracted with 1 mL of acidified water (0.2 N HCl). After centrifugation, the absorbance of the developed pink-colored solution was measured at 520 nm. The percentage of pyocyanin reduction in the presence of nanometals-treated cultures was determined and compared to the control untreated ones.

##### Elastase Activity Assay

The elastase action of cell-free supernatants of *P. aeruginosa* PAO-JP2, PAO1, PAO14, KU6, and 52 which were cultured with and without Au-NPs and Se-NPs at sub-MICs (1/2 and 1/4 MICs) was carried out using the elastin Congo red (ECR, Oxoid, Basingstoke Hants, UK) assay [96]. Aliquots of each culture supernatant (500 μL) were incubated with an equal volume of ECR buffer (1.22% Tris/HCl) containing 20 mg/mL of ECR and incubated with shaking at 37 °C for 16–18 h. The mixture was then centrifuged and the absorbance of soluble ECR was recorded at 495 nm. The percentage reduction in elastolytic action of treated in comparison to untreated cultures was estimated.

##### Total Protease Assay

A skimmed milk assay was used to assess the proteolytic activity of *P. aeruginosa* isolates [97]. *P. aeruginosa* supernatants treated with sub-MICs of Au-NPs and Se-NPs were incubated with 1 mL of 1.25% (*w*/*v*) skimmed milk for 30 min at 37 °C and the OD600 nm was measured. Control cultures without Au-NPs and Se-NPs were simultaneously assessed. Compared to untreated isolates, the decrease in the proteolytic effect of Au-NPs and Se-NPs-treated isolates was determined via an increase in the absorbance of the reading.

### 4.9. Real-Time PCR

RT-PCR was used to measure the effect of biologically prepared Au-NPs and Se-NPs on the expression of QS regulatory genes *lasIR*/*rhlIR* and PQS in *P. aeruginosa* PAO1 and virulence factors (*lasB* and *toxA*). Total RNA of PAO1 treated with 1/2 MIC of Au-NPs and Se-NPs was extracted using TRI reagent following the manufacturer’s instructions (Oxoid, Basingstoke Hants, UK) [14]. Complementary DNA was synthesized using the SensiFAST™ cDNA Synthesis Kit (Bioline Reagents Ltd., London, UK). RT-PCR was carried out via the thermocycler Rotor-Gene Q (Qiagen, Valencia, CA, USA). The amplification reaction was set up using TOPreal™ qPCR 2X PreMIX (SYBR Green with low ROX), (Enzynomics; Daejeon, Korea) utilizing primers listed in Table 2. The level of each gene’s expression was normalized to the expression of the *rpoD* housekeeping gene, and the relative expression was calculated as 2^−^^ΔΔCT^ [98]. Fold changes in gene expression in PAO1 cultivated with the prepared nanometals were compared to its expression in the control cultures without treatment.

### 4.10. Statistical Analysis

The data were represented as an average value obtained from three independent set-ups. A Student’s *t* test (*p* < 0.05) was applied to analyze significant differences between control and treated values propagated under the same conditions

## 5. Conclusions

In this work, we showed the synthesis of Au-NPs and Se-NPs using cell free-*Streptomyces* S91 supernatant. Specifically, Au-NPs were more effective than Se-NPs against the tested *Pseudomonas* strains. The MIC of the prepared Au-NPs was in the range of 9.2–147.7 µg/mL and for Se-NPs was in the range of 4.6–1184.4 µg/mL against the tested *Pseudomonas* strains. Furthermore, the synthesized nanometals possessed excellent anti-QS and antivirulent properties with potent activity of Au-NPs compared to Se-NPs. Eventually, both nanometals significantly inhibited the production of pyocyanin, elastase, and protease from the tested *P. aeruginosa* strains, unequivocally demonstrated their wide spectrum anti-QS properties. Hence, the biosynthesized Au-NPs and Se-NPs could be valuable in nanotechnology applications since they could be used as nano-anti-QS and virulent inhibitors for the control of *P. aeruginosa* acute and chronic infections. Further studies, stability, cytotoxicity and in vivo activities will be performed to assess the antipathogenic effect of the prepared nanometals.

## Figures and Tables

**Table 1:** The minimum inhibitory concentrations (MICs)/minimum bactericidal concentrations (MBCs) and sub-inhibitory concentrations (1/2 and 1/4 MICs) of the biosynthesized nanometals against *P. aeruginosa* isolates.

	*P. aeruginosa*	MICs /MBCs of NPs (µg/mL)	1/2 MIC (µg/mL)	1/4 MIC (µg/mL)
Au-NPs	PAO-JP2	9.2/18.5	4.6	2.3
	PAO1	36.9/147.7	18.5	9.2
	PAO14	18.5/36.9	9.2	4.6
	KU6	18.5/147.7	9.2	4.6
	52	18.5/73.9	9.2	4.6
Se-NP	PAO-JP2	4.6/74.02	2.3	1.2
	PAO1	592.2/1184.4	296.1	148.1
	PAO14	592.2/1184.4	296.1	148.1
	KU6	592.2/1184.4	296.1	148.1
	52	1184.4/1184.4	592.2	296.1
	PAO-JP2	˂2/˂2	˂2	˂2
Ceftazidime	PAO1	4/16	2	1
	PAO14	64/128	32	16
	KU6	16/128	8	4
	52	4/8	2	1
	PAO-JP2	˂2/˂2	˂2	˂2
	PAO1	16/128	8	4
	PAO14	8/2048	4	2
Ciprofloxacin	KU6	256/256	128	64
	52	256/512	128	64

**Table 2:** Specific amplification primer sets used in RT-PCR.

Primer	Sequence (5′→3′)	Annealing Temperature (°C)	Amplicon (bp)
RpoD For	CGAACTGCTTGCCGACTT	56	131
RpoD Rev	GCGAGAGCCTCAAGGATAC		
LasI For	CGCACATCTGGGAACTCA	56	176
LasI Rev	CGGCACGGATCATCATCT		
LasR For	CTGTGGATGCTCAAGGACTAC	56	133
LasR Rev	AACTGGTCTTGCCGATGG		
RhlI For	GTAGCGGGTTTGCGGATG	58	101
RhlI Rev	CGGCATCAGGTCTTCATCG		
RhlR For	GCCAGCGTCTTGTTCGG	56	160
RhlR rev	CGGTCTGCCTGAGCCATC		
PqsA For	GACCGGCTGTATTCGATTC	58	74
PqsA rev	GCTGAACCAGGGAAAGAAC		
PqsR For	CTGATCTGCCGGTAATTGG	56	142
PqsR rev	ATCGACGAGGAACTGAAGA		
LasB For	GGTAGAACGCACGGTTGT	56	165
LasB rev	GGCAAGAACGACTTCCTGAT		
ToxA For	CCGCCGAAGACGATGCTT	58	85
ToxA rev	CACCGCCAACTGGAGGAG		

## Supplementary Materials

The following are available online at www.mdpi.com/article/10.3390/antibiotics10121461/s1, Table S1: Distribution of *Streptomyces* strains isolated from different soil samples, Table S2: UV-Vis spectroscopy analysis wavelength range of 300–1000 nm of cell-free supernatant of *Streptomyces* isolates S1, SS2, S91, S29B and S41.1 untreated and treated with HAuCl_4_ and SeO_2_. Table S3: Inhibition of violacein pigment production by *C. violaceum* ATCC 12472 using biogenic nanometals. Figure S1: Effect of sub-inhibitory concentrations as detected by crystal violet method (a) control untreated cultures (b) 1/2 and (c) 1/4 MICs of Au-NPs and (c) 1/2 and (d) 1/4 MICs of Se-NPs on biofilm formation by *P. aeruginosa* PAO1, PAO14 compared to control untreated cultures.
