# Supplementary material for: Inhibition of Quorum Sensing and Virulence Factors of Pseudomonas aeruginosa by Biologically Synthesized Gold and Selenium Nanoparticles
Source: Antibiotics (Basel). 2021 Nov 27;10(12):1461. doi: 10.3390/antibiotics10121461 (PMC8698379; doi:10.3390/antibiotics10121461)
Supplement: Supplementary file 1 [file antibiotics-10-01461-s001.zip › antibiotics-1458472-supplementary.pdf]

Inhibition of Quorum sensing and Virulence Factors of *Pseudomonas aeruginosa* by Biologically Synthesized Gold and Selenium Nanoparticles

Soha Lotfy Elshaer, Mona I. Shaaban*

Department of Microbiology and Immunology, Faculty of Pharmacy, Mansoura University, 35516, Egypt

***Corresponding author**
Mona Shaaban
Department of Microbiology and Immunology, Faculty of Pharmacy, Mansoura University, Egypt
Email; mona_ibrahem@mans.edu.eg

Table S1. Distribution of *Streptomyces* strains isolated from different soil samples.

Location	Type of soil samples	Longitude	Latitude	Number of soil samples	*Streptomyces* characters		
					White	Grey	Total
1.Dakahlia	Cultivated	31.381523	31.037933	6	5	11	16
2.Damietta	Sandy	31.814444	31.417540	2	3	6	9
Total				8	8	17	25

Table S2: UV–Vis spectroscopy analysis wavelength range of 300–1000 nm of cell-free supernatant of *Streptomyces* isolates S1, SS2, S91, S29B and S41.1, untreated and treated with $HAuCl_4$ and SeO_2.

Salt Conc.	Time (hours)	Spectrum 300-1000 nm	Cell free supernatant	S1	SS2	S91	S29B	S41.1
**Se (60mM)**	**48**	**λ** (A)	**400** (0.155)	-ve	-ve	**450 (1.1)**	-ve	-ve
**Au (3mM)**	**48**	**λ** (A)	**673** (0.237)	**641** (0.177)	**583** (0.594)	**520** (1.172)	**536** (2.054)	**582-590** (1.78)

Table S3: Inhibition of violacein pigment production by *Chromobacterium violaceum* ATCC 12472 by biogenic NPs.

Biogenic NPs	QSI diameter (mm)
**Au-NPs**	11
**Se-NPs**	7.5

QSI; Quorum Sensing Inhibition, NPs; nanoparticles

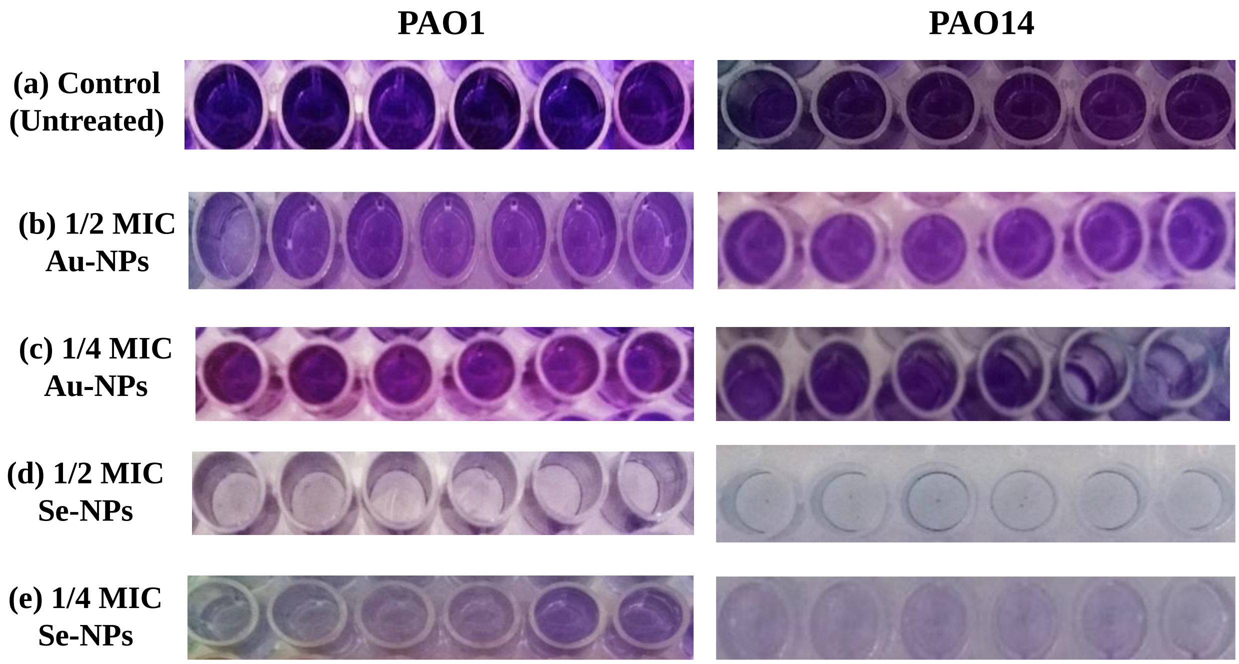

Figure. S1

Effect of sub-inhibitory concentrations as detected by crystal violet method(**a**) control untreated cultures (**b**) 1/2 and (c) 1/4 MICs of Au-NPs and (**c**) 1/2 and (**d**) 1/4 MICs of Se-NPs on biofilm formation by *P. aeruginosa* PAO1, PAO14 compared to control untreated cultures.
